# Performance Characteristics of a High-Throughput Automated Transcription-Mediated Amplification Test for SARS-CoV-2 Detection

**DOI:** 10.1128/JCM.01669-20

**Published:** 2020-09-22

**Authors:** Jimmykim Pham, Sarah Meyer, Catherine Nguyen, Analee Williams, Melissa Hunsicker, Ian McHardy, Inessa Gendlina, D. Yitzchak Goldstein, Amy S. Fox, Angela Hudson, Paul Darby, Paul Hovey, Jose Morales, James Mitchell, Karen Harrington, Mehrdad Majlessi, Joshua Moberly, Ankur Shah, Andrew Worlock, Marion Walcher, Barbara Eaton, Damon Getman, Craig Clark

**Affiliations:** a Hologic, Inc., San Diego California, USA; b Scripps Health, Microbiology Laboratory, San Diego California, USA; c Montefiore Medical Center, Department of Pathology, Bronx, New York, USA; Marquette University

**Keywords:** SARS-CoV-2, automation, high throughput, Aptima, TMA

## Abstract

The COVID-19 pandemic caused by the new SARS-CoV-2 coronavirus has imposed severe challenges on laboratories in their effort to achieve sufficient diagnostic testing capability for identifying infected individuals. In this study, we report the analytical and clinical performance characteristics of a new, high-throughput, fully automated nucleic acid amplification test system for the detection of SARS-CoV-2. The assay utilizes target capture, transcription-mediated amplification, and acridinium ester-labeled probe chemistry on the automated Panther system to directly amplify and detect two separate target sequences in the open reading frame 1ab (ORF1ab) region of the SARS-CoV-2 RNA genome. The probit 95% limit of detection of the assay was determined to be 0.004 50% tissue culture infective dose (TCID_50_)/ml using inactivated virus and 25 copies/ml (c/ml) using synthetic *in vitro* transcript RNA targets. Analytical sensitivity (100% detection) was confirmed to be 83 to 194 c/ml using three commercially available SARS-CoV-2 nucleic acid controls. No cross-reactivity or interference was observed with testing of six related human coronaviruses, as well as 24 other viral, fungal, and bacterial pathogens, at high titers. Clinical nasopharyngeal swab specimen testing (*n* = 140) showed 100%, 98.7%, and 99.3% positive, negative, and overall agreement, respectively, with a validated reverse transcription-PCR nucleic acid amplification test (NAAT) for SARS-CoV-2 RNA. These results provide validation evidence for a sensitive and specific method for pandemic-scale automated molecular diagnostic testing for SARS-CoV-2.

## Abstract (precis)

The COVID-19 pandemic caused by the new SARS-CoV-2 coronavirus has imposed severe challenges on laboratories in their effort to achieve sufficient diagnostic testing capability for identifying infected individuals. In this study, we report the analytical and clinical performance characteristics of a new, high-throughput, fully automated nucleic acid amplification test system for the detection of SARS-CoV-2. The assay utilizes target capture, transcription-mediated amplification, and acridinium ester-labeled probe chemistry on the automated Panther system to directly amplify and detect two separate target sequences in the open reading frame 1ab (ORF1ab) region of the SARS-CoV-2 RNA genome.

## INTRODUCTION

Coronavirus disease-19 (COVID-19) is a novel respiratory illness caused by severe acute respiratory syndrome coronavirus 2 (SARS-CoV-2), a novel *Sarbecovirus* that emerged from the region of Wuhan, China, in late 2019 (1). People with COVID-19 experience mild to severe respiratory symptoms, including fever, cough, and shortness of breath or difficulty breathing (2), although some individuals experience no symptoms at all (3).

The COVID-19 pandemic has occurred across all continents, adding more than 100,000 new SARS-CoV-2 cases globally each day (4, 5). As communities begin reopening and relaxing quarantine measures, there is the potential risk for an upsurge in cases and rates of viral transmission. The availability of validated high-throughput diagnostic tests is therefore essential for rapidly and efficiently informing patient management decisions, implementing hospital infection prevention practices, and guiding public health responses to wide-scale infection control measures to reduce transmission in populations.

To meet the need for pandemic-scale diagnostic testing, we developed and validated a high-throughput, fully automated nucleic acid amplification test (NAAT) for direct amplification and detection of SARS-CoV-2 RNA from specimens of infected individuals. The assay employs target capture, transcription-mediated amplification (TMA), and acridinium ester-labeled probe chemistries to enable a sample-to-result solution for detection of two different conserved target regions within the open reading frame 1ab (ORF1ab) region of the SARS-CoV-2 genome. Here, we describe the analytical and clinical performance characteristics of the assay.

## MATERIALS AND METHODS

### Transcription-mediated amplification test for SARS-CoV-2.

The Aptima SARS-CoV-2 assay utilizes magnetic bead-based target capture, isothermal TMA of RNA, and dual kinetic acridinium ester-labeled probe hybridization for the isolation, amplification, and detection of an internal process control RNA and two unique sequences within the ORF1ab region of the SARS-CoV-2 viral genome. The assay is performed on the automated Panther and Panther Fusion instruments (both from Hologic, Inc., San Diego, CA) and received FDA emergency use authorization (EUA) on 14 May 2020. It is intended for the qualitative detection of SARS-CoV-2 RNA isolated and purified from nasopharyngeal (NP) swab, nasal swab (NS), midturbinate and oropharyngeal (OP) swab, NP wash/aspirate, or nasal aspirate specimens obtained from individuals meeting COVID-19 clinical and/or epidemiological criteria. The sample input volume is 0.5 ml with continuous sample and reagent loading access and automated RNA extraction, amplification, detection, and result reporting. The time to first result is 3 h and 30 min, with a capacity of approximately 1,025 results per 24 h per instrument system.

### Comparison assay.

The validated EUA Panther Fusion SARS-CoV-2 reverse transcription-PCR (RT-PCR) assay (Hologic, Inc.) was used as a comparator assay for clinical performance studies. This assay was performed as previously described (6).

### Analytical performance.

**(i) Limit of detection.** The analytical sensitivity of the SARS-CoV-2 TMA assay was assessed using 2 lots of reagents to test 60 replicates each of dilution panels containing cultured SARS-CoV-2 virus strain USA-WA1/2020 (BEI Resources, Manassas, VA) and diluted in Aptima specimen transport medium (STM) matrix to a range of 0.03 to 0.0003 50% tissue culture infectious dose (TCID_50_)/ml. Also tested were replicates (*n* = 43 to 60) of panels consisting of two *in vitro* transcribed (IVT) RNA targets, corresponding to two unique target sequences within the ORF1ab region of the SARS-CoV-2 RNA genome, diluted in STM. The assay positivity for both studies was determined using a cutoff value of 560 kilo relative light units (kRLU) according to the manufacturer’s instructions for use. Results were analyzed by probit analysis (normal model) to determine the 95% limit of detection (LOD). Analytical sensitivity was confirmed by diluting stock SARS-CoV-2 virus in STM into four specimen matrices (pooled NP swab specimens, STM, saline, and liquid Amies transport medium (Copan, Murrieta, CA) at 0.003 TCID_50_/ml for NP swab, STM, and saline and 0.003 and 0.01 TCID_50_/ml for liquid Amies (*n* = 20 replicates for each determination).

**(ii) Analytical specificity/interference.** Analytical specificity of the SARS-CoV-2 TMA assay was determined by evaluating assay cross-reactivity and interference using 30 nontarget microorganisms (17 viral species, including 6 non-SARS-CoV-2 coronaviruses, 11 bacterial species, and 2 fungal species; *n* = 3 replicates each) at the highest titer achievable. Thirty NP swab specimens obtained from asymptomatic donors (with consent) were also tested to represent diverse microbial flora in the human respiratory tract. Interference by high-titer nontarget organisms was assessed by testing 3 replicates of each organism in the presence of low-titer (0.03 TCID_50_/ml) SARS-CoV-2 virus. Cross-reactivity by high-titer nontarget organisms was assessed in the absence of SARS-CoV-2 virus.

**SARS-CoV-2 commercial control panel testing.** Stock SARS-CoV-2 control panel materials from three commercial suppliers (Exact Diagnostics [Fort Worth, TX] SARS-CoV-2 standard, catalog no. COV019; SeraCare [Milford, MA] AccuPlex SARS-CoV-2 verification panel, catalog no. 0505-0126; and ZeptoMetrix [Buffalo, NY] SARS-CoV-2 external run control, catalog no. NATSARS[COV2]-ERC0831042) were diluted in stock STM to 6 concentrations ranging from 8 to 833 copies per ml (c/ml), and multiple replicates (*n* = 20 to 40) were tested with the SARS-CoV-2 TMA assay. The control panels were also tested with the SARS-CoV-2 RT-PCR assay on the automated Panther Fusion platform.

### Specimen collection and clinical performance.

Residual deidentified NP swab samples were collected using standard methods from 140 symptomatic patients at two U.S. clinical sites (Salt Lake City, UT, and Albuquerque, NM). Specimens were transported to the laboratory and tested with the SARS-CoV-2 TMA assay and the Fusion SARS CoV-2 RT-PCR assay. An additional clinical sample set consisting of paired NP swab, OP swab, and NS specimens were collected from 38 patients; complete sets (containing all three specimen types) were obtained from 35 patients. NP swabs were collected using either BD universal viral transport nasopharyngeal swab and universal viral transport medium (VTM) (Becton, Dickinson, San Diego, CA) or Copan Minitip flocked swab and VTM. NS samples were collected first by inserting the swab into the subject’s nostril past the inferior turbinate, approximately 3 cm, twisting the swab in the midturbinate area for 3 to 5 s, and placing the swab into a tube of STM. OP swab samples were collected immediately following NS samples by swabbing the posterior pharynx for 3 to 5 s and placing the swab into a specimen tube containing STM. Samples were frozen and shipped to Hologic for testing.

## RESULTS

**FIG 1:**
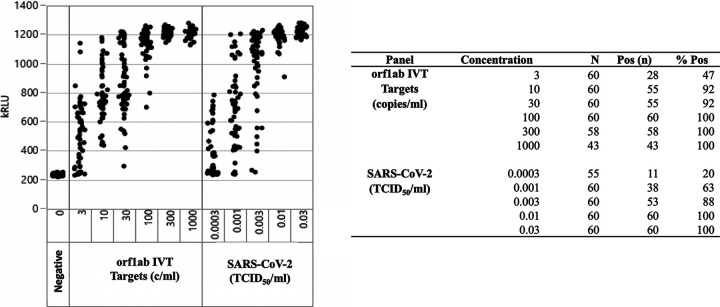
Analytical sensitivity of automated Aptima SARS-CoV-2 TMA assay for detection of SARS-CoV-2 open reading frame 1ab (ORF1ab) RNA. IVT, *in vitro* RNA transcript; TCID_50_, 50% tissue culture infectious dose.

Data for the analytical sensitivity determination of the SARS-CoV-2 TMA assay are shown in Fig. 1. Using a predetermined cutoff value of 560 kRLU, the assay yielded 100% positivity at a concentration of 0.01 TCID_50_/ml of SARS-CoV-2 virus and at 100 c/ml of SARS-CoV-2 IVT RNA targets. Using probit analysis, the 95% limit of detection was determined to be 0.004 TCID_50_/ml (95% confidence interval [CI], 0.003 to 0.007) for SARS-CoV-2 virus, and 25.4 c/ml (95% CI, 16.9 to 50.5) for ORF1ab IVT RNA targets. Analytical sensitivity for the assay was confirmed by testing SARS-CoV-2 virus in 4 specimen matrices (pooled NP swab specimens, STM, saline, and liquid Amies transport medium) at 0.003 TCID_50_/ml. All specimen matrices yielded 95% positivity (19/20 replicates detected) at this concentration, except the liquid Amies transport medium, which was 85% (17/20) positive at 0.003 TCID_50_/ml and 100% (20/20) positive at 0.01 TCID_50_/ml (Table 1).

**TABLE 1:** Confirmation of Aptima SARS-CoV-2 TMA assay limit of detection in different specimen matrices^*a*^

Target	Matrix	TCID_50_/ml	*n*/*N* (%)	Avg kRLU (% CV)
SARS-CoV-2 virus	NP	0	0/0 (0)	278 (2.9)
		0.003	19/20 (95)	908 (17.2)
	STM	0	0/0 (0)	289 (2.2)
		0.003	19/20 (95)	835 (24.4)
	Saline	0	0/0 (0)	288 (2.4)
		0.003	19/20 (95)	876 (24.8)
	Liquid Amies	0	0/0 (0)	286 (1.8)
		0.003	17/20 (85)	877 (24.7)
		0.01	20/20 (100)	1100 (3.9)

a STM, Aptima specimen transport medium; *n*/*N*, number positive/number tested; kRLU, kilo relative light unit; CV, coefficient of variation.

The analytical specificity of the SARS-CoV-2 TMA assay was determined by evaluating assay cross-reactivity and interference using 30 nontarget viral, bacterial, and fungal microorganisms at the highest titer achievable, as well as in 30 NP swab specimens obtained with consent from asymptomatic donors at low risk of SARS-CoV-2 infection. As shown in Table S1 in the supplemental material, none of the microorganisms or NP swab specimens tested caused cross-reactivity in the absence of SARS-CoV-2 target or interfered with TMA detection in the presence of SARS-CoV-2 spiked at 0.03 TCID_50_/ml.

**FIG 2:**
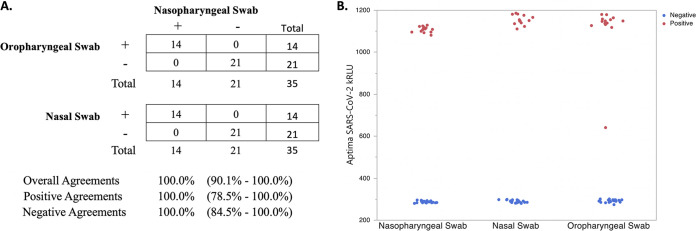
(A) Agreement between 35 sets of co-collected nasopharyngeal swab, oropharyngeal swab, and nasal swab clinical specimens with positive (+) and negative (–) Aptima SARS-CoV-2 TMA assay results. (B) Scatterplot of corresponding Aptima SARS-CoV-2 TMA assay positive and negative kRLU signal for each sample type.

The clinical accuracy of the SARS-CoV-2 TMA assay was compared to the SARS-CoV-2 RT-PCR assay using 140 patient NP swab specimens (Table 2, Table S2). This analysis resulted in positive, negative, and overall agreements of 100% (95% CI, 94.3% to 100%), 98.7% (95% CI, 92.9% to 99.8%), and 99.3% (95% CI, 96.1% to 99.9%), respectively. Clinical performance of the SARS-CoV-2 TMA assay was also assessed by testing sets of NP swab, OP swab, and nasal swab specimens co-collected from 35 symptomatic patients suspected of being infected with SARS-CoV-2. Figure 2 shows that the SARS-CoV-2 TMA assay had 100% positive and negative agreements of the NP swab specimens with the co-collected OP swab and nasal swab specimens. Similar results were obtained for the paired specimen sets using the SARS-CoV-2 RT-PCR assay (Fig. S1).

**TABLE 2:** Agreement analysis between the Aptima SARS-CoV-2 TMA assay and the Panther Fusion SARS-CoV-2 RT-PCR assay for nasopharyngeal swab specimens^*a*^

Aptima SARS-CoV-2 TMA result	Panther Fusion SARS-CoV-2 RT-PCR assay result		
	Positive	Negative	Total
Positive	64	1	65
Negative	0	75	75
Total	64	76	140

a Overall agreement was 99.3% (95% CI, 96.1% to 99.9%), positive agreement was 100% (95% CI, 94.3% to 100%), and negative agreement was 98.7% (95% CI, 92.9% to 99.8%).

SARS-CoV-2 control panel materials from three commercial suppliers (Exact Diagnostics, SeraCare, and ZeptoMetrix) were evaluated by building dilution panels of each control material and testing multiple replicates with both the SARS-CoV-2 TMA assay and the SARS-CoV-2 RT-PCR assay (Table 3). Both assays yielded similar results. For the Exact Diagnostics SARS-CoV-2 control, the TMA and RT-PCR assays each had 100% detection down to 83 c/ml (*n* = 40 each). For the SeraCare SARS-CoV-2 control, the TMA assay was 100% at 83 c/ml (*n* = 20), while the RT-PCR assay was 90% at 83 c/ml and 100% at 194 c/ml (*n* = 20 for both). The SARS-CoV-2 TMA assay (*n* = 37) and the SARS-CoV-2 RT-PCR assay (*n* = 40) were both 100% reactive at 194 c/ml using the ZeptoMetrix control material.

**TABLE 3:** Performance of Aptima SARS-CoV-2 TMA and Panther Fusion SARS-CoV-2 RT-PCR assays for detection of commercially available SARS-CoV-2 controls^*a*^

SARS-CoV-2 control vendor	Panther Fusion SARS-CoV-2 RT-PCR result		
	Concn (c/ml)	Aptima SARS-CoV-2/TMA (*n*/*N*) (%)	Panther Fusion SARS-CoV-2 RT-PCR (*n*/*N*) (%)
Exact Diagnostics SARS-CoV-2 standard	833	40/40 (100)	38/38 (100)
	417	40/40 (100)	39/39 (100)
	194	40/40 (100)	40/40 (100)
	83	**40/40 (100)**	**40/40 (100)**
	19	35/39 (90)	30/40 (75)
	8	29/39 (74)	21/40 (53)
SeraCare Accuplex SARS-CoV-2 verification panel	833	20/20 (100)	20/20 (100)
	417	19/19 (100)	20/20 (100)
	194	19/19 (100)	**20/20 (100)**
	83	**20/20 (100)**	18/20 (90)
	19	16/40 (40)	11/40 (28)
	8	7/40 (18)	4/40 (10)
ZeptoMetrics SARS-CoV-2 (recombinant)	833	39/39 (100)	40/40 (100)
	417	40/40 (100)	40/40 (100)
	194	**37/37 (100)**	**40/40 (100)**
	83	39/40 (97.5)	32/40 (80)
	19	12/40 (30)	14/40 (35)
	8	12/40 (30)	8/40 (20)

a The lowest concentration of each panel with 100% positivity is shown in bold type. *n*/*N*, number positive/number tested.

## DISCUSSION

The availability of large-scale diagnostic testing for SARS-CoV-2 addresses a current critical need for identifying the prevalence and spread of the virus in populations and for guiding public health policies and interventions to minimize incident infections. This study describes the performance characteristics of a new high-throughput isothermal TMA NAAT that employs a complete sample-to-result automation system to maximize sample testing throughput in clinical laboratories. The analytical performance data demonstrate that the assay is highly sensitive and specific for detection of viral SARS-CoV-2 RNA and has high clinical agreement (100% positive agreement, 98.7% negative agreement) with an EUA-validated RT-PCR assay for SARS-CoV-2 RNA.

The unprecedented need for SARS-CoV-2 testing has resulted in national shortages in sample collection materials, including VTM, prompting the CDC and FDA to recommend optional specimen collection media, such as saline and liquid Amies (7, 8). To ensure comparable performance across various collection media, we evaluated the analytical sensitivity of the SARS-CoV-2 TMA assay in NP swab matrix (NP swabs collected in VTM), STM, saline, and liquid Amies transport medium using inactivated SARS-CoV-2 virus. NP swab matrix, Aptima STM, and saline demonstrated 95% detection at 0.003 TCID_50_/ml, whereas liquid Amies showed slightly lower sensitivity at 0.01 TCID_50_/ml. Despite this small difference in analytical sensitivity, these results suggest that all of these media are acceptable for use for clinical sample collection.

NP swab specimens have been considered the gold standard sampling method for respiratory virus infection (9), as it has been reported that nasal swab or OP swab samples may have a slightly lower sensitivity than NP swab samples (10, 11). However, given the challenges of collection device shortages for SARS-CoV-2 diagnosis, particularly NP swabs and VTM, the use of nasal swab and OP swab as alternate samples for diagnosis of SARS-CoV-2 has been evaluated. The CDC recently removed NP swab as the “preferred” sample type from their sample collection guidelines (7), and others have reported comparable performance of NS and OP swabs for the diagnosis of SARS-CoV-2 (12–15). Our data show strong agreement between SARS-CoV-2 detection from NS and OP swab samples compared to paired NP swab samples for the SARS-CoV-2 TMA and SARS-CoV-2 RT-PCR assays. For one patient, we did observe positive NP and nasal swab results but negative results in the paired OP swab; however, overall, the data indicate that both NS and OP swab specimens are adequate sample types for diagnosis of SARS-CoV-2.

The performance of other SARS-CoV-2 NAATs that have received emergency use authorization has been characterized using commercially available inactivated virus preparations (e.g., BEI Resources, Manassas, VA) or synthetic RNA quality control materials. Our evaluation of three different external RNA control materials demonstrated comparable analytical sensitivity with both the SARS-CoV-2 TMA assay and the SARS-CoV-2 RT-PCR assay, with LOD values of between 83 c/ml of 194 c/ml. These analytical sensitivity values correlate with previously reported LOD values by Smith et al. (16). However, using LOD values as determined by external control material to assess or compare assay performance warrants some caution, as different control materials may give considerably different results for the absolute LOD value (16), and the reported RNA or DNA stock concentrations of these materials may differ from true concentrations (17).

The limitations of this study include the small number of clinical specimens available for testing and the absence of discordant result resolution by testing with a third assay. The paired specimen testing (NS and OP) included only 35 patients (14 of which were positive). The specimens that were included did span a typical clinical titer range for the virus (SARS-CoV-2 RT-PCR cycle threshold [*C_T_*] values ranged from 14.5 to 37.1). Additional clinical data should be collected to more fully assess and compare performance between these sample types. We also only tested control panel material in STM, not in patient swab specimen matrix.

In summary, the SARS-CoV-2 TMA assay is highly sensitive and specific and provides an automated high-throughput testing solution for large-scale diagnostic testing for the virus. The assay system is able to process and generate results for >1,000 samples per day, enabling medical centers, reference laboratories, and public health laboratories to efficiently process and analyze very high volumes of specimens for the detection of SARS-CoV-2 RNA (18).

## Supplementary Material

Supplemental file 1
