# Supplementary material for: Performance Characteristics of a High-Throughput Automated Transcription-Mediated Amplification Test for SARS-CoV-2 Detection
Source: J Clin Microbiol. 2020 Sep 22;58(10):e01669-20. doi: 10.1128/JCM.01669-20 (PMC7512162; doi:10.1128/JCM.01669-20)
Supplement: Supplemental file 1 [file JCM.01669-20-s0001.pdf]

A.

		Nasopharyngeal Swab +	Nasopharyngeal Swab -	Total
**OroPharyngeal Swab**	+	13	0	13
	-	1	21	22
	Total	14	21	35

		Nasopharyngeal Swab +	Nasopharyngeal Swab -	Total
**Nasal Swab**	+	14	0	14
	-	0	21	21
	Total	14	21	35

Overall Agreements	97.1%	(85.5% - 99.5%)
Positive Agreements	92.9%	(68.5% - 98.7%)
Negative Agreements	100.0%	(84.5% - 100.0%)

B.

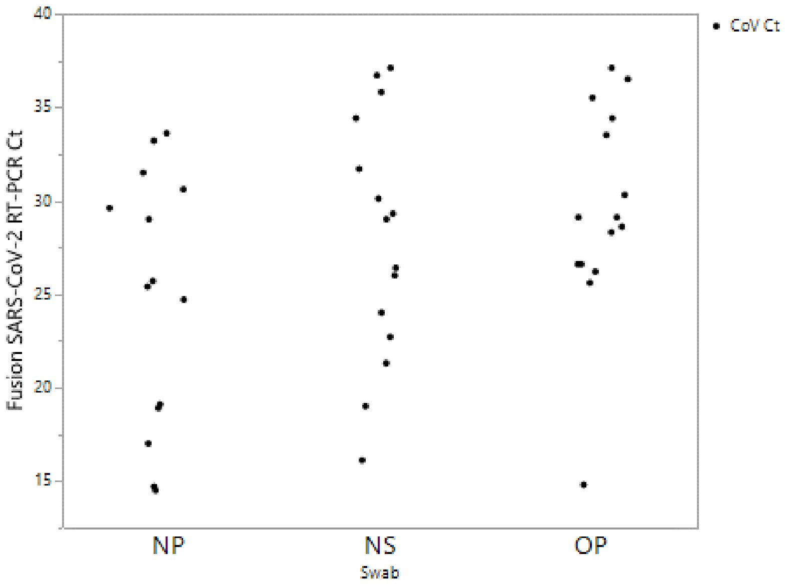

Supplemental Figure. Agreement (**A**) between 35 sets of co-collected nasopharyngeal swab, oropharyngeal swab, and nasal swab clinical specimens with positive (+) and negative (-) Panther Fusion SARS-CoV-2 RT-PCR assay results. Scatter plot (**B**) of RT-PCR Ct values corresponding to Panther Fusion SARS-CoV-2 RT-PCR assay positive samples for each swab type.

Supplemental Table 1. Microorganism cross reactivity and interference of the Aptima SARS-CoV-2 TMA assay.

		SARS-CoV-2 Unspiked			SARS-CoV-2 Spiked [a]		
**Microorganism**	**Concentration**	**N Tested**	**N Detected**	**% Detected**	**N Tested**	**N Detected**	**% Detected**
No organism Control	N/A	3	0	0	3	3	100
Human coronavirus 229E	1.00E+05 TCID50/ml	3	0	0	3	3	100
Human coronavirus OC43	1.00E+05 TCID50/ml	3	0	0	3	3	100
Human coronavirus HKU1	1.00E+06 c/ml	3	0	0	3	3	100
Human coronavirus NL63	1.00E+04 TCID50/ml	3	0	0	3	3	100
SARS-coronavirus	1.00E+06 c/ml	3	0	0	3	3	100
MERS-coronavirus	1.00E+04 TCID50/ml	3	0	0	3	3	100
Adenovirus	1.00E+05 TCID50/ml	3	0	0	3	3	100
Human Metapneumovirus (hMPV)	1.00E+06 TCID50/ml	3	0	0	3	3	100
Parainfluenza virus 1	1.00E+05 TCID50/ml	3	0	0	3	3	100
Parainfluenza virus 2	1.00E+05 TCID50/ml	3	0	0	3	3	100
Parainfluenza virus 3	1.00E+05 TCID50/ml	3	0	0	3	3	100
Parainfluenza virus 4	1.00E+03 TCID50/ml	3	0	0	3	3	100
Influenza A (H3N2)	1.00E+05 TCID50/ml	3	0	0	3	3	100

		SARS-CoV-2 Unspiked			SARS-CoV-2 Spiked [a]		
**Microorganism**	**Concentration**	**N Tested**	**N Detected**	**% Detected**	**N Tested**	**N Detected**	**% Detected**
Influenza B	2.00E+03 TCID50/ml	3	0	0	3	3	100
Enterovirus (e.g. EV68)	1.00E+05 TCID50/ml	3	0	0	3	3	100
Respiratory syncytial virus	1.00E+05 TCID50/ml	3	0	0	3	3	100
Rhinovirus	1.00E+04 TCID50/ml	3	0	0	3	3	100
Chlamydia pneumonia	1.00E+06 IFU/ml	3	0	0	3	3	100
Haemophilus influenzae	1.00E+06 CFU/ml	3	0	0	3	3	100
Legionella pneumophila	1.00E+06 CFU/ml	3	0	0	3	3	100
Mycobacterium tuberculosis	1.00E+06 TCID50/ml	3	0	0	3	3	100
Streptococcus pneumonia	1.00E+06 CFU/ml	3	0	0	3	3	100
Streptococcus pyogenes	1.00E+06 CFU/ml	3	0	0	3	3	100
Bordetella pertussis	1.00E+06 CFU/ml	3	0	0	3	3	100
Mycoplasma pneumoniae	1.00E+06 CFU/ml	3	0	0	3	3	100
Pneumocystis jirovecii	1.00E+06 nuc/ml	3	0	0	3	3	100
Candida albicans	1.00E+06 CFU/ml	3	0	0	3	3	100
Pseudomonas aeruginosa	1.00E+06 CFU/ml	3	0	0	3	3	100

		SARS-CoV-2 Unspiked			SARS-CoV-2 Spiked [a]		
**Microorganism**	**Concentration**	**N Tested**	**N Detected**	**% Detected**	**N Tested**	**N Detected**	**% Detected**
Staphylococcus epidermidis	1.00E+06 CFU/ml	3	0	0	3	3	100
Streptococcus salivarius	1.00E+06 CFU/ml	3	0	0	3	3	100
Negative clinical NP swab specimens (N=30[b])	N/A	90	0	0	90	90	100

[a]SARS-CoV-2 inactivated cultured virus spiked at 0.03 $TCID_{50}$/ml (3x LoD)

[b]Each NP swab specimen was tested in triplicate for a total of 90 replicates in the absence and presence of spiked SARS-CoV-2 inactivated virus.

Supplemental Table 2. Line listing of results from Table 2 for 140 nasopharyngeal swab specimens tested with the Aptima SARS-CoV-2 assay and the Panther Fusion SARS-CoV-2 assay.

**Sample ID**	**Aptima SARS-CoV-2**		**Panther Fusion SARS-CoV-2**	
	**RLU (x1000)**	**Result**	**Ct**	**Result**
1	1164	POS	29.1	POS
2	303	NEG	0	NEG
3	282	NEG	0	NEG
4	297	NEG	0	NEG
5	284	NEG	0	NEG
6	1163	POS	15	POS
7	1140	POS	18.1	POS
8	309	NEG	0	NEG
9	295	NEG	0	NEG
10	1105	POS	30.5	POS
11	1142	POS	20.9	POS
12	299	NEG	0	NEG
13	1140	POS	26.2	POS
14	296	NEG	0	NEG
15	1105	POS	15.7	POS
16	1132	POS	24.4	POS

17	297	NEG	0	NEG
18	300	NEG	0	NEG
19	294	NEG	0	NEG
20	1182	POS	32.1	POS
21	297	NEG	0	NEG
22	297	NEG	0	NEG
23	306	NEG	0	NEG
24	300	NEG	0	NEG
25	296	NEG	0	NEG
26	299	NEG	0	NEG
27	303	NEG	0	NEG
28	303	NEG	0	NEG
29	1166	POS	19.8	POS
30	1187	POS	0	NEG
31	1165	POS	30.8	POS
32	297	NEG	0	NEG
33	1187	POS	28	POS
34	1172	POS	32.4	POS
35	1107	POS	30.9	POS
36	303	NEG	0	NEG
37	314	NEG	0	NEG
38	311	NEG	0	NEG
39	297	NEG	0	NEG
40	1182	POS	29.7	POS
41	306	NEG	0	NEG
42	1108	POS	21.9	POS
43	294	NEG	0	NEG
44	295	NEG	0	NEG
45	286	NEG	0	NEG
46	291	NEG	0	NEG
47	283	NEG	0	NEG
48	287	NEG	0	NEG
49	1184	POS	21.8	POS
50	1171	POS	24.1	POS
51	289	NEG	0	NEG
52	1192	POS	29.4	POS
53	1192	POS	23.2	POS
54	1196	POS	22.2	POS
55	1204	POS	24.7	POS
56	1165	POS	31.2	POS
57	287	NEG	0	NEG
58	288	NEG	0	NEG
59	290	NEG	0	NEG

60	288	NEG	0	NEG
61	284	NEG	0	NEG
62	275	NEG	0	NEG
63	285	NEG	0	NEG
64	294	NEG	0	NEG
65	1182	POS	22.3	POS
66	288	NEG	0	NEG
67	1176	POS	30.8	POS
68	1154	POS	22.3	POS
69	296	NEG	0	NEG
70	295	NEG	0	NEG
71	304	NEG	0	NEG
72	1155	POS	17	POS
73	292	NEG	0	NEG
74	1187	POS	19.7	POS
75	1207	POS	22.5	POS
76	283	NEG	0	NEG
77	1181	POS	18.3	POS
78	295	NEG	0	NEG
79	294	NEG	0	NEG
80	1231	POS	31.1	POS
81	290	NEG	0	NEG
82	285	NEG	0	NEG
83	303	NEG	0	NEG
84	299	NEG	0	NEG
85	1228	POS	27.1	POS
86	288	NEG	0	NEG
87	272	NEG	0	NEG
88	1203	POS	19.5	POS
89	1189	POS	16.3	POS
90	1130	POS	20	POS
91	1167	POS	33.1	POS
92	1139	POS	17.5	POS
93	1126	POS	24.2	POS
94	1140	POS	24.3	POS
95	1154	POS	16	POS
96	1107	POS	19.8	POS
97	1127	POS	33.6	POS
98	1147	POS	20.7	POS
99	1116	POS	22.2	POS
100	1152	POS	17.8	POS
101	1129	POS	24	POS
102	1186	POS	24.2	POS

103	1105	POS	21.8	POS
104	1168	POS	35.3	POS
105	1169	POS	25.6	POS
106	283	NEG	0	NEG
107	284	NEG	0	NEG
108	285	NEG	0	NEG
109	289	NEG	0	NEG
110	293	NEG	0	NEG
111	288	NEG	0	NEG
112	283	NEG	0	NEG
113	285	NEG	0	NEG
114	290	NEG	0	NEG
115	292	NEG	0	NEG
116	282	NEG	0	NEG
117	284	NEG	0	NEG
118	280	NEG	0	NEG
119	287	NEG	0	NEG
120	291	NEG	0	NEG
121	1123	POS	33.2	POS
122	1121	POS	33.6	POS
123	1096	POS	29.6	POS
124	1100	POS	19.1	POS
125	1111	POS	25.4	POS
126	1116	POS	18.9	POS
127	1129	POS	14.7	POS
128	1125	POS	25.7	POS
129	1111	POS	30.6	POS
130	1109	POS	14.5	POS
131	290	NEG	0	NEG
132	283	NEG	0	NEG
133	296	NEG	0	NEG
134	283	NEG	0	NEG
135	295	NEG	0	NEG
136	283	NEG	0	NEG
137	1081	POS	29	POS
138	1096	POS	17	POS
139	1107	POS	31.5	POS
140	1093	POS	24.7	POS
